# The role of teachers in a sustainable university: from digital competencies to postdigital capabilities

**DOI:** 10.1007/s11423-023-10199-z

**Published:** 2023-02-06

**Authors:** Lina Markauskaite, Lucila Carvalho, Tim Fawns

**Affiliations:** 1 Sydney School of Education and Social Work, The University of Sydney, Education and Social Work Building A35, Camperdown, NSW 2006 Australia; 2 Massey University, Auckland, New Zealand; 3 Monash Education Academy, Monash University, Melbourne, Australia

**Keywords:** Teaching capabilities, Teacher competencies, An ecological perspective, Postdigital science, The good university, Sustainable university, Sustainable development

## Abstract

An increase in online and hybrid education during and after the Covid-19 pandemic has rapidly accelerated the infiltration of digital media into mainstream university teaching. Global challenges, such as ecological crises, call for further radical changes in university teaching, requiring an even richer convergence of ‘natural,’ ‘human’ and ‘digital’. In this paper, we argue that this convergence demands us to go beyond ‘the great online transition’ and reframe how we think about university, teachers’ roles and their competencies to use digital technologies. We focus on what it takes to be a teacher in a sustainable university and consider emerging trends at three levels of the educational ecosystem—global developments (macro), teachers’ local practices (meso), and daily activities (micro). Through discussion of examples of ecopedagogies and pedagogies of care and self-care, we argue that teaching requires a fluency to embrace different ways of knowing and collective awareness of how the digital is entwined with human practices within and across different levels of the educational ecosystem. For this, there is a need to move beyond person-centric theorisations of teacher digital competencies towards more holistic, ecological conceptualisations. It also requires going beyond functionalist views of teachers’ roles towards enabling their agentive engagement with a future-oriented, sustainable university mission.

## Introduction: capabilities for teaching in universities that do not exist yet

‘Online teaching’, ‘distance teaching’, ‘e-learning’, and other modes of teaching with digital technologies, once seen as a distinct area of teachers’ competence, have become inextricably mixed with other modes of teaching—’hybrid’, ‘blended’, and ‘hyflex’, to mention a few (Brown, 2016; Trede et al., 2019). An emerging line of scholarship on postdigital education further questions if we can make distinctions between digital and non-digital modalities of learning and teaching when much of our educational practices fuse both (Fawns, 2019; Jandrić et al., 2018), particularly since ‘the great online transition’, which forced us to dissolve many dichotomies between online and on-campus teaching (MacKenzie et al., 2021).

Further, back in 2015, the United Nations launched the Sustainable Development Goals as a universal call to action to secure peace and prosperity, ensure quality education, protect the planet, and end poverty for all by 2030 (United Nations, 2015). Accordingly, a number of universities have been reflecting on what these goals entail for them (SDSN, 2020). Some scholars have been arguing that it is time for radical change, emphasising the need for identifying core values and outlining visions of a sustainable, ‘good university’ (Bengtsen & Gildersleeve, 2022; Connell, 2019; Facer, 2019). With technologies enmeshed in many aspects of teaching and learning, and indeed, in many aspects of everyday living, it is crucial that the digital is no longer seen as detached from global challenges and our visions of what universities ought to be (Goodyear, 2022; Nørgård, Mor, & Bengtsen, 2019). This is rarely reflected in our thinking about teachers’ digital competencies. Existing syntheses of research tend to highlight benefits and limitations of different ways of understanding university teachers’ competencies to teach with digital technologies (cf., Albrahim, 2020; Baran et al., 2011; Cutri & Mena, 2020; Goodyear et al., 2001; Muñoz Carril et al., 2013; Natividad Beltrán del Río, 2021), but there is very little discussion about what underpins contextualised teaching in which the use of digital technologies is an inseparable part of a broader future-oriented goal and mission.

In this paper, we address these issues through one central question: *What does it take to be a teacher in a future-oriented university in which* ‘natural,’ ‘human,’ and ‘digital’ are inextricably enmeshed? We argue that there is an urgent need to reconsider teachers’ digital competencies, proposing an expansion of thinking along four lines: (1) from the neoliberal university to the sustainable university; (2) from digital to postdigital; (3) from competencies to capabilities; and (4) from teacher personal resourcefulness to distributed teaching capabilities.

Our argument is developed through three moves. First, we develop the foundations for our perspective by elaborating on the four lines above. Next, we draw on contemporary postdigital approaches that embrace ecopedagogies, pedagogies of care and self-care to ground our propositions in emerging pedagogical movements and explore what postdigital teaching capabilities might entail within university contexts. Finally, we bring these insights together to foreground the importance of teachers’ fluency to embrace different ways of knowing and their collective awareness of how the digital is entwined with human practices across the educational ecosystem. As a part of this we argue that two fundamental shifts are critical. First, there is a need to move beyond person-centric theorisations of teacher digital competencies towards more holistic, ecological conceptualisations. Second, there is a need to go beyond functionalist views of teachers’ roles towards enabling their agentive engagement with a future-oriented, sustainable university mission. We offer this paper as an entry point for engaging university teachers and other practitioners with this broader role and transformative mission.

## Foundational perspectives

### Rethinking university: towards a sustainable university

Universities play a key role in the ongoing, sustainable functioning of society. They generate much of the scientific and technical knowledge that underpins economic progress and social change; they prepare professionals for most complex public services (e.g., health, law) and industries (e.g., IT, banking); and they help us think creatively and critically about history, culture, philosophy, and the arts (Connell, 2019).

Universities emerged in an era of information scarcity when access to knowledge was limited and intended for only a small elite—often people of economic means, male and white. Throughout their history, universities have been supported by underlying mechanisms that reinforce power and control, producing and reproducing certain privileged values and knowledge practices while neglecting others, promoting certain academics over others, and supporting certain students while excluding or marginalising others (Boys, 2022; Connell, 2019). The last century has brought the rise of neoliberalism with its market-oriented economic and social reforms and policies. This has also influenced the ways higher education prepares professionals by emphasising skills and attitudes for a productive and profitable workforce rather than a broader set of values (Connell, 2019).

Increased access to knowledge and information through open digital platforms has brought the role and function of universities under scrutiny. Consequently, the current university system, and its teaching practices, are perceived to be in crisis or, at least, falling short of their potential (Connell, 2019). Some scholars urge universities to embrace a new mission and values in all aspects of their functioning (Bengtsen & Gildersleeve, 2022; Connell, 2019; Facer, 2019; Goodyear, 2022). For example, Connell (2019) describes the following qualities of a good university and their implications for teaching:

- *Democratic:* values and ways of working that relate to the university’s ability to serve “democratic purposes of the society” and place “social justice… [as a] core business for the teaching programme” (p. 171–172).
- *Engaged*: “being fully present for the society that supports the university”, where good teaching “means being fully present for the students, engaging with their actual needs and enabling their next moves in learning” (p. 172). Connell notes that this is demanding for teachers in relation to time, emotion and technical knowledge.
- *Truthful:* doing research in a truthful way and engaging in more epistemically transparent teaching practices that “emphasise how knowledge is produced and archived, helping students to test claims, challenge received knowledge, and conduct their own investigations” (p. 173).
- C*reative:* foregrounding students’ agency and teachers’ imagination, offering “space for wilderness in classrooms, mad professors, and educational risk-taking—knowing that only some of them will succeed” (p. 174).
- *Sustainable:* the university’s organisation and functioning, including employment conditions, support all workers over time. This must be within the society’s “resource limits” (p. 174) and contribute to the sustainable knowledge economy as a whole by “building knowledge commons in the world at large” (p. 174).

Connell’s proposal allows us to envisage teaching capabilities that are needed for universities to contribute more meaningfully to the sustainable development of society.^1^ For change to occur, university staff, and particularly teachers, need to be agents who think broadly and whose actions matter (Bengtsen & Gildersleeve, 2022; Goodyear, 2022; Nørgård & Bengtsen, 2021). As we discuss next, this involves an engagement with digital technology as integrated within a broader ecosystem.

### Rethinking digital: the postdigital perspective

The notion of postdigital foregrounds digital technologies as part of a heterogeneous entanglement in education, as part of wider social, epistemic, material, and spatial structures, acting at multiple scale levels, from individual activities to megatrends and cultures. A postdigital perspective rejects viewing the digital as separated from material and social activity as digital information, education, networks, and technologies are always embedded in the world (Fawns, 2019; Jandrić et al., 2018). For example, online teaching can help with access issues for particular people, including some who find it difficult to physically attend at specific times and places, but it can make different things harder or easier for different people (Czerniewicz & Carvalho, 2022).

Online, hybrid, or any teaching that involves digital technologies, often have been associated with teachers’ digital capabilities. Through postdigital lenses, teaching and learning are seen as part of complex configurations of human and non-human actors, as an assemblage of elements that extends far beyond single physical classroom settings, specific digital tools and material elements, or pedagogical practices, towards a range of interconnected elements, such as government policy, university strategy, digital technologies and others (Lamb et al., 2022). For example, the unfamiliar and challenging context of a recent global pandemic informed which methods of teaching and assessment were appropriate (e.g., online) while encouraging reliance on particular technologies (e.g., Zoom) and shaping what was pedagogically possible and feasible.

A postdigital perspective opens new avenues for thinking about the role of technology in education. It can help us to understand connections at multiple dimensions and scale levels—from classrooms, to learning tasks, to curricula, to policy, to wider infrastructure, to broader community, to the environment and so on (Carvalho & Yeoman, 2018). As such, forms of digital education—online, blended or hybrid—are enmeshed into the material, social, cultural, political, economic, and environmental fabrics of society. This perspective encourages us to move beyond individualistic conceptions of human actors, such as teachers, and beyond apparently human-centric activities, such as teaching. Technology is understood as social and material, and so too, is teaching. This postdigital perspective leads to a reframing of what is meant by teacher competencies to teach with digital technologies.

### Rethinking competencies: a capability approach

Over decades, there have been numerous discussions about the meaning of terms that describe a human’s ability to complete particular tasks, such as ‘proficiency’, ‘competency’, ‘competence’, ‘capability’, and ‘expertise’ (Eraut, 1998; Markauskaite & Goodyear, 2017; OECD, 2019). Such terms are often understood as broad, as encompassing “knowledge, skills and attitudes (beliefs, dispositions, values)” (OECD, 2019, p. 99), with little agreement on what each term means, how they differ and how they relate to each other. The literature in adult and professional learning tends to use ‘competency’ to refer to functional capacities to perform particular tasks in real-life contexts. For example, an explanation in the OECD Survey of Adult Skills asserts that:

“Competency is the capacity to generate appropriate performance: to marshal the resources (tools, knowledge, techniques) in a social context (which involves interacting with others, understanding expectations) to realise a goal that is appropriate to the context.” (OECD, 2019, p. 99)

‘Competence’ (plural ‘competences’) is often considered to be a more holistic term than ‘competency’ (plural competencies), with the latter seen as constituents of the former (Blömeke et al., 2015). Competence is also often described in a normative sense as “the ability to perform tasks and roles to the *expected standard*” (Eraut, 1998, p. 127, emphasis added). Here, the emphasis is on meeting expectations of others, which implies that “its precise meaning [is] to be negotiated by stakeholders in a macro- or micro-political context” (p. 127).

Eraut (1998) distinguishes between ‘competence’ and ‘capability’, arguing that ‘capability’ is a broader term that includes ‘competence’. Capability refers to “everything a person can think or do, given an appropriate context for demonstrating it” and is “individually situated and profession referenced” (Eraut, 1998, p. 135). In contrast, competence is “socially situated and job referenced” (p. 135). Most importantly, if competence is related to demonstrated performance, then capability is related to one’s potential, and it is oriented towards future performance. This open-endedness of the term ‘capability’ makes it suitable for discussions about the future. However, the growing need to address sustainable development challenges in education, requires moving beyond individually situated and profession referenced conceptualisations.

Sen’s (1999) capability approach, sometimes adopted in the professional literature (Poquet & de Laat, 2021; Sandars & Sarojini Hart, 2015), is particularly helpful here. According to Sen, capabilities are connected to people’s freedoms to be and to do what they value so that they can achieve these values. In contrast to person-centred definitions of competences, such as those adopted in psychology (Blömeke et al., 2015), Sen’s capabilities are foregrounded as more than a person’s individual abilities, or the absence of constraints, instead as encompassing the *actual opportunities* that help people to achieve their values. This includes freedoms to pursue moral responsibilities, but it also requires constraining oneself and reconciling personal values, values of the profession, and values of the society. This view of capabilities recognises a diversity of values and the complexity of contexts that teachers ought to navigate in present times. It emphasises a relational agency to make choices that are appropriate for themselves, for other people, and for the environment, and to *enact* these choices.

Sen’s conception enables us to broaden our thinking about university teachers’ digital capabilities as connected to actual freedoms and opportunities, and the need to reconcile the values of different actors and stakeholders through teaching practices. This requires a further shift in focus: from individual teacher to broader, distributed activity system.

### Rethinking teaching: an activity systems view

Most of the literature takes a person-centred view, which considers competencies or capabilities as personal attributes, something teachers possess independently of situation and context. However, teaching is often enacted collectively by people and technologies (Dron, 2021). Students, for example, may be intimately implicated in collective acts of teaching, and so too may be learning technologists, learning designers, administrators, managers, IT staff, and librarians, in addition to policymakers, employers, accreditors, and so on.

Drawing on recent research (Reimann & Markauskaite, 2023; Stigler & Miller, 2018), we take an activity systems’ view towards capabilities to foreground teaching as a distributed activity. In so doing, our focus is on ‘teaching capabilities’ rather than ‘teacher capabilities’, to explicitly acknowledge that teaching and capabilities to teach include the distributed agency of multiple educational stakeholders and are influenced by a wide web of elements which include curricula, educational policies, leadership, joint goals and visions, disciplines, physical and digital resources, socio-political contexts, natural environment, etc.

In short, teachers can play a key role in transformative action in society, but in order to work towards realising visions of the good university, it is important to widen our thinking about teaching capabilities with digital technologies. These capabilities span global (macro), local (meso), and individual (micro) levels of the educational ecosystem and are intertwined with what we can call ‘postdigital pedagogies’, to which we turn next.

## Teaching and postdigital pedagogies

There is no lack of ideas in educational literature on what future curricula and pedagogical approaches may look like—pedagogies of hope, ecopedagogies, passion curricula, slow pedagogies, humanising pedagogies, university activism, etc. (Bengtsen & Gildersleeve, 2022; Miziaszek, 2020; Nørgård et al., 2021). However, there has been little discussion about what these pedagogical movements entail for teaching capabilities. It may not seem that digital technologies are a central concern here, but the digital is firmly entwined with what is happening in, for, and with the world (Nørgård, Mor, & Bengtsen, 2019). Given the ecological and humanitarian crises, how can we embrace technologies in a purposive and sustainable way? How do we rethink teaching capabilities? In this section, we explore ecopedagogies, and pedagogies of care and self-care. We chose pedagogies that are primarily associated with macro (the planet), meso (university environment) and micro (embodied self) of the educational ecosystem in which teachers work to offer grounding for deeper insights into their implications for university teaching capabilities.

### Ecopedagogies

Ecopedagogies have their roots in transformation-based teaching models (Miziaszek, 2020) and can be associated with early ideas from critical theory (Freire, 1972) and critiques of educational practices (Illich, 1983). Freire (1972) saw literacy as connected to people’s ability to ‘read the world’, advocating for the need to empower people to creatively and critically deal with reality to help them figure out how to best transform their own world. Ecopedagogies go one step further to highlight that ‘reading the world’ is intrinsically connected to ‘reading our planet Earth’; and, as Jandrić and Ford (2022) argue,

“The Earth that ecopedagogy reads is postdigital, and the literary practices and technologies we use to engage in such generative reading are implicated in new geopolitical and social realities” (p. xiv).

As such, ecopedagogies include education models aimed at ending socio-environmental injustices and violence (Gadotti, 2011; Miziaszek, 2020), including those that we see in our digital infosphere (Jandrić & Ford, 2022). These models search for deeper understandings of who benefits and who suffers from actions that are harmful to the environment, foregrounding that some suffer more than others. Ecopedagogies invite combinations of multiple perspectives, as well as different knowledges and ways of knowing (e.g., indigenous and western knowledge). They suggest the adoption of a holistic view whilst asking for a shift in mindset from local to global, to understand issues from a planetary perspective, and to act to transform societal structures.

Digital technologies play a crucial role in enacting these pedagogical ideas by facilitating connections and the emergence of new kinds of learning communities, where people can engage in discussions of different perspectives and participate in processes of knowledge co-creation (Networked Learning Editorial Collective, 2021). Yet, ecopedagogical models encourage teachers and students to go deeper, and to consider the role of digital technologies beyond their immediate instrumental purposes. These include complex ethical entanglements, such as taking account of not only potential pedagogical benefits of videoconferencing, artificial intelligence (AI) and other power-intensive digital technologies but also their consumption of natural resources and their broader impact on the environment (Knox, 2019). Another example is the importance of considering inequalities that may emerge at different levels, such as challenges of access and use of technologies (Czerniewicz & Carvalho, 2022). Ecopedagogies involve teachers unpacking hidden assumptions related to development and sustainability, which might include not only helping students learn to use technology but also when not to use it or when to find alternatives. In short, the role of digital technologies in education can be broadly seen in relation to other areas of life and development, and teachers play a key role in facilitating such discussions with their students.

From a transformational perspective, teaching and learning also needs to go beyond dialogue around complex relations between local and global, to include capabilities to reimagine, co-create, and take future action. For teachers, this involves more than designing content or tasks, but making space to go beyond the ‘normal’ and expected (Boys, 2016), and engaging students and other people in the mutual negotiation of values and social priorities (Taylor & Bovill, 2018). Ecopedagogies ask teachers and students to be open and flexible, to search for the value of multiple perspectives, to take different disciplinary stances into account, and to be inclusive of different types of knowledge and ways of knowing. Such teaching and learning are inseparable from engagement in distributed communities, use of multiple knowledge sources and other ways of knowing intertwined with digital technologies.

### Pedagogies of care

A recent convergence of crises (e.g., Covid-19, climate change, the war in Ukraine) highlights the urgency of caring for each other, our bodily conditions, our systems and societies. A caring perspective has also been gaining momentum in higher education, calling for commitment to ethics, empathy and social justice (Mehta & Gleason, 2021; Morel, 2021; Motta & Bennett, 2018). If education is to be ‘truthful’ (Connell, 2019), it must confront challenging and inconvenient issues (e.g., decolonisation; diversity and inclusivity; hierarchies and power dynamics).

What teaching capabilities are needed for enacting care in such an environment? Noddings (1984, 2002) proposes that care is founded on reciprocal relations between carer and cared for, characterised by listening, receptiveness, and presence. For Noddings, caring teachers do not impose their own values, ideas, and principles onto learners, and do not base their actions on assumptions about students. Instead, they are receptive to what each student articulates about what they do, need, are, and want to become. This might require deviating from prescribed course, programme, or institutional goals, and being open to alternative paths and possibilities. For example, rather than helping a student achieve better marks, it may be more important to help them choose a different subject, or to leave University altogether. Thus, pedagogies of care can be in tension with common values and priorities of teachers and neoliberalist institutions, whose focus is often on graduating students that are ready for the job market.

While pedagogies of care emphasise teacher-student relations, educational activity is distributed more widely (Rose & Adams, 2014). Distributed care involves attending to the relations between all elements in a learning ecology. In other words, we cannot produce caring or socially just education simply by adding care or social justice to individual interactions. It quickly becomes clear that this work cannot be done by individuals and that collective action is required at different levels of the institution at the micro, meso, and macro levels. In developing teaching capabilities for enacting pedagogies of care and social justice, teachers must develop a capacity for collective action. This challenge is, perhaps, most readily apparent in relation to moving classes online during the Covid-19 pandemic (Green et al., 2020; Hodges et al., 2020; Williamson et al., 2020). Online tools were initially adopted at scale and speed, often without sufficient and focused learning design to ensure inclusive participation. Enacting an ethic of care included considering differences in students’ technological access and home infrastructures, what happens with students’ data, or how technology is implicated in trust and community-building (Bali & Zamora, 2022).

Care in higher education is particularly challenging in relation to AI and other automatic technology. If, for example, teachers allow technology to project its reality onto students (e.g., where learning analytics constrain legitimate behaviour or knowledge), then we cease to be in what Noddings (1984, 2002) would characterise as a caring relationship. Yet, technologies do not act independently towards or against values, such as care. Rather, their influence works through situated entanglements with purposes, values, contexts, and teaching methods (Fawns, 2022). For example, technologies can also be used to engage with wider communities and gather alternative perspectives, or allow for creative possibilities and a broader variety of options for assessment, tasks, group work, etc. As Noddings (1984) argues, caring is not about hierarchical or universal principles, or logic, but curiosity, contact, connection, and empathy. Students live real, enmeshed, domestic, familial, working and studying lives, struggling to find the time and the space to study. An important teaching capability might include intentionally designing and orchestrating educational tasks that consider technology, not in isolation but in relation to different facets of student life, including mental health, equity in assessment, access to resources, physical and digital accessibility, and the inclusion of different, legitimate forms of expression of knowledge.

### Pedagogies of self-care

In considering the wider educational ecosystem and how it is sustained, it is worth asking whether teachers can enact a pedagogy of care (Noddings, 1984, 2002) without caring for, or being cared for, themselves (Rose & Adams, 2014). Care, like respect and trust, is reciprocal (Ladson-Billings, 1995). Covid-19 made us aware that teacher emotional states and responses could be critical when they shift to teaching and learning in digital environments (Owens & Hudson, 2021).

Some teacher self-care is also necessary for sustained contribution to the distributed expertise of the system, and to avoid personal cares becoming burdens that prevent care for others (Noddings, 1984). Yet, caring for the self is only possible through caring for others, and collective caring is only possible when those involved have sufficient resources (Bali & Zamora, 2022; Noddings, 1984). Therefore, self-care is linked to a broader ethic of care that involves both a system that allows space, time and energy to care, and individuals knowing their own limits and values (since knowing oneself provides a basis for understanding the realities of others, Noddings, 2002).

**Fig. 1:**
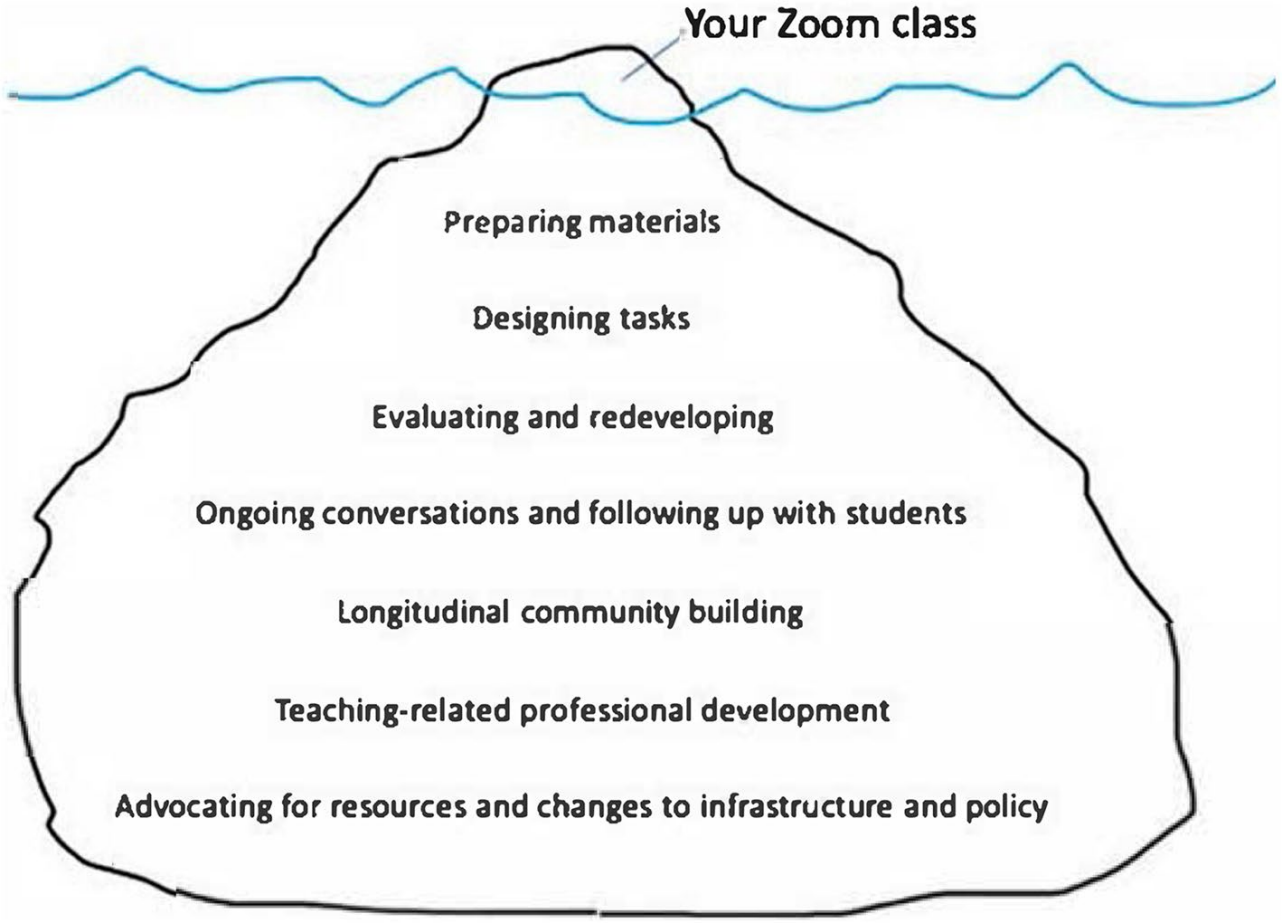
The online teaching iceberg (Fawns et al., 2021b, p. 226)

This can be challenging within systems that constrain our capacity to think through the implications of actions, and to reflect on what matters to us, our colleagues, and our students. Part of teaching capability may, therefore, involve making space for teacher wellbeing, thinking about teaching, constructively challenging systems and cultures, and recognising otherwise invisible labour and the expertise that accompanies it. Fawns et al. (2021) observe the example of videoconferencing sessions as just the tip of the iceberg of what teachers do. Much more time and effort are spent below the surface, and much more is involved in developing associated teaching capabilities (Fig. 1).

The further we dive below the surface, the more recognition of these activities as part of teaching diminishes. Advocating for resources and changes to infrastructure and policy, for example, is crucial to inclusivity and social justice, yet is unlikely to be recognised in workload allocation models or formal teaching evaluations.

Overall, teaching often involves complex and uncertain situations, which are likely to require sustained and, often, invisible work, away from the simpler and more obvious concerns of teaching sessions. This is particularly challenging where different values are in tension (e.g., inclusivity is often at odds with rhetoric of efficiency), and in the context of a fast-moving educational landscape in which new technologies are continuously introduced and abandoned. As Bussey (2021) notes, integrating technology into the various educational and administrative practices of teaching can place considerable pressure on teachers. The ‘digital capability’ of educators also includes recognition of when a complicated technology is unnecessary, as a simple one would do.

Further, effective teaching in hybrid spaces is a complex, skilled activity that inevitably involves multitasking, carries extreme cognitive load, and needs to be performed publicly in front of students under pressure and stress (MacKenzie et al., 2021; Raes et al., 2020). For example, during Covid-19, teachers and others tried to manage fatigue and pressure, and figure out how to adapt design and practice such that they themselves, their students and others could cope in unfamiliar and unstable contexts. Self-care requires embracing “human biology and cognition into the same assemblage of digital education as values, policy, digital technologies, learning spaces, and voices of students and teachers” (MacKenzie et al., 2021, p. 304). It involves going beyond simplistic conceptions of online or hybrid teaching capabilities as following ‘teaching tips’ or ‘best practices’, to enabling teachers’ understanding of deeper principles of complex skilful human performance, including what causes cognitive load and stress, and how to reduce it by redesigning teaching and learning environments.

In short, self-care involves educators re-examining the purposes and values of education, and developing knowledge, skills and strategies to prioritise what matters, and caring for themselves and others in sustainable ways. It relies upon, and calls for, trust in the wider educational ecosystem at micro, meso and macro levels.

## Discussion

### Postdigital pedagogies and capabilities

Each of the pedagogical perspectives discussed above foregrounds different but complementary levels of the educational ecosystem. The sustainability-oriented stance of ecopedagogies encourages a global (macro) orientation, while pedagogies of care and self-care are primarily focused on the local (meso and micro) environment and the situated self. Nevertheless, all three perspectives focus on relations, and emphasise connections across the educational ecosystem. Thus, drawing strict lines between them would be a fundamental mistake. There is nothing at the micro level, without the meso and the macro instantiations. There is no self-care without caring for others and for the environment, and vice versa.

Postdigital teaching capabilities, therefore, are distributed and relational. They involve engagement in a range of practices and performances. Conceptualising such capabilities requires that we revisit the foundations of human capability for complex performance.

Classical models of expertise that look at how people become good at what they do often adopt person-centric information processing views of human cognition and suggest that repetitive deliberate practice and mastery of routines are key (Ericsson, 2006). However, Reimann and Markauskaite (2023) argue that this is insufficient in contemporary teaching contexts. Firstly, teaching requires *adaptive expertise* (Bohle Carbonell et al., 2014) as it involves not only mastering routines but also the continuous development of knowledge and skills, particularly when teaching with digital technologies. Secondly, teaching requires *distributed expertise* (Hutchins, 1995; Salomon, 1993), as teaching is performed not in the head, but in the world. It is inseparable from embodied, situated performance that intertwines personal mental resourcefulness with the material and the social environments of the activity. Further, teachers increasingly need to work in teams with other professionals, such as learning designers, IT managers, student support staff, and future employers. Therefore, they need *relational expertise* to recognise what kinds of complementary capabilities other people bring and how they can be combined (Edwards, 2010; Hakkarainen et al., 2017). Finally, teaching is also a highly complex professional domain that involves specialised forms of knowledge and requires *expertise to co-create* diverse professional knowledge products. This includes instructional resources, theories of action, design principles, and other kinds of principled and actionable knowledge that can be shared with the teaching team or profession (Bereiter, 2013; Markauskaite & Goodyear, 2017).

In short, teaching involves relationships between embodied self, environment, other people and professional knowledge, with professional capability going far beyond what individuals possess, to include cognitive, material, social and epistemic dimensions. This requires broadening current ways of conceptualising teaching capabilities in hybrid environments by adopting more holistic, ecological lenses.

### Postdigital teaching capabilities through ecological lenses

Over 20 years ago, Goodyear et al., (2001) observed that online teacher competencies are usually conceptualised either from a pragmatic *competence-based perspective* that aims to produce detailed normative lists of what kinds of knowledge and other personal attributes teachers ought to have, or from a *cognitive perspective* that aims to go one step beyond these lists to describe knowledge structures and mental processes that underpin skilful performance. There are also those who take a *humanistic perspective* to teaching, and often object to reducing human capability to any explicit list or model, and instead focus on teachers’ agency, vulnerability and identity (Cutri & Mena, 2020).

However, useful conceptualisations of teaching capabilities cannot rely on models that see teachers’ capabilities entirely as properties inherent in an individual, be they ‘skull-bound’ cognitive competencies or products of individual agency. Useful conceptualisations must describe the capabilities that extend across an activity system which includes other people, culture (norms), environment and self as an embodied and vulnerable being. As Säljö (2010) argued:

“We cannot look for human competencies solely in our minds or bodies. Instead, our knowledge is expressed in our abilities to merge and collaborate with external tools and integrate them into the flow of our doings, whether intellectual, physical or mixed.” (p. 62)

Indeed, theories of human cognition that look at human performance and learning in complex real-world environments are increasingly moving beyond the classical person-focussed information processing accounts of cognition which previously dominated conceptualisations of professional competencies and deliberate practice. They see human capabilities as extended, enacted, embedded, enculturated and embodied (Hutchins, 2010; Markauskaite, 2020; Markauskaite & Goodyear, 2017). Such grounded ecological models do not dismiss the importance of individual human minds and skills but acknowledge that what humans know and are capable of doing cannot be separated from their feelings, embodied experiences, actions, their material and digital environments, historical contexts, and social others.

Ecological models enable us to shift the focus from digital competencies as primarily being characteristics of individuals and their solo performance towards a more distributed view of capabilities that situates individual performance within a larger distributed activity system (Trede et al., 2019). They help us reframe what teachers need to know, and what roles they should be able to take, foregrounding teachers’ capabilities to recognise and make connections between different kinds of knowledge, the encountered environment and what matters in an unfolding activity. Such teaching capabilities require that teachers are not only competent in a particular area, but, even more importantly, are fluent in recognising, switching between and integrating different perspectives and ways of knowing. This includes ways of knowing that are intertwined with digital technologies.﻿

Drawing on this perspective, Markauskaite and Goodyear (2017) identify four key dimensions of such fluency: (1) combination and integration of different kinds of knowledge; (2) coordination and weaving different ways of knowing; (3) creating tools and assembling productive environments; and (4) constructing a conscientious and conscious self (Markauskaite & Goodyear, 2017; Trede et al., 2019). These dimensions connect well to postdigital pedagogies and can facilitate insight into what could underpin capabilities for teaching in hybrid environments.

Firstly, real-life cases provide strong evidence that teachers’ knowledge and capabilities that enable them to teach, in practice, come in different forms and from different experiences, including formal professional learning and everyday personal encounters (Kali et al., 2011; Markauskaite & Goodyear, 2014). There is a need for flexible models that account for integration of diverse knowledge resources on which teachers draw in practice, when teaching with digital technologies. This diversity might expand further when considering classroom teaching in the context of ecopedagogies and pedagogies of care. Some of this knowledge could be formally acquired—one may read about what ecopedagogy means. Some could be tacit—one may gain knowledge by ‘reading the world’, such as by working alongside students listening to online lectures in overcrowded university learning spaces and close-by shopping malls. Framing digital technologies neither as separate from macro-level concepts, nor disconnected from situated teaching and learning experiences requires *drawing on and integrating different kinds of knowledge*.

Secondly, working with different kinds of knowledge also requires embracing different ways of knowing. Ecopedagogies, pedagogies of care and self-care and others cannot work without expanding teachers’ understanding of different ways in which distributed teaching and learning can be made productive and how they can draw on different ways of knowing and learning when they teach. This might involve knowing when it is best to spend time designing a handout to support students’ equitable participation in online discussions and when to engage in deliberate practice to fine-tune personal skills to use Zoom and to orchestrate groupwork; when to learn certain things yourself and when to work alongside with more knowledgeable colleagues; when to make decisions about students learning yourself and when to outsource part of this work to learning analytics tools. Such decisions reverberate not only by changing contexts but are also inseparable from teachers’ flexibility to recognise what makes teaching productive in a particular situation. This recognition cannot be achieved through explicit professional learning alone and requires teachers to engage in different kinds of expert practices, whilst *weaving different ways of knowing*.

Thirdly, sustainable, caring or socially just, and other more distributed teaching practices are not done simply by engaging in complex social interactions. Tools and environments connected to those practices also play an essential role. For example, scholars point out to the importance of embracing empathic design in digital learning environments (Morel, 2021). Others suggest centring on the lived realities of learners by engaging learners in co-design (Mehta & Gleason, 2021). More equitable learning might also require a shift from face-to-face and synchronous teaching towards co-creating access to shared knowledge resources and aligning practices among members of the teaching team, non-teaching professionals and students. Such practices are impossible without a significant shift from direct teaching to teaching as co-design (Goodyear, 2015) to *co-create tools and assemble productive environments* that allow sustainable, caring, socially just distributed practices.

Fourthly, responsive collective practices and actions are impossible without attention to individuals and attunement to others, the environment and the self. Attunement involves the ability to notice and take action whilst aligning one’s responses to various visible and invisible states and the actions of others. Being conscientious means being aware and attuned to others, including humans and other living organisms, the natural environment and digital agents. Being conscious means being aware and attuned to one’s own knowledge, skills, feelings, and wellbeing. These are essential elements of distributed capabilities for teaching. Professionals, including teachers, are increasingly surrounded by digital tools that extend their natural senses (e.g., learning analytics), including self-tracking tools (e.g., daily screen time), and these tools have increasingly become intertwined with teacher abilities to care for self, for others and for our environment. There is plenty of evidence to be optimistic about the potential of digital tools to support the values of ‘a good university’ as well as for being cautious about the power of these tools to distort these values (Williamson et al., 2020). *Being conscious and conscientious* about how digital tools intertwine with our natural senses and values, and figuring out how to use them in ways that enhance our actions in the classroom, university, professional community and the world, is a key teaching capability in a postdigital university.

## Conclusions

As complex global challenges escalate, there is urgent need for changes in education. Universities can play a crucial role in finding ways to address major social, ecological and humanitarian issues. As universities ponder how to meaningfully contribute to sustainable development goals, Misiaszek (2020) warns us that a key concern here relates to grasping what ‘development’ actually means, that is, to recognise the need to de-emphasise its connection to economic models and neoliberal agendas, and to see the local as part of a larger ecosystem. It is also important to note that ‘the university’ is an entity populated by people, including teachers, whose actions matter and who can be crucial agents for enabling transformation in society.

Thus, a fundamental teaching capability is teachers’ awareness of how the digital is entwined with human practices within and across different levels of the educational ecosystem and fluency to navigate and co-create ‘postdigital learning ecologies’. Teachers need to traverse divisions between knowledge domains, and ways of knowing, and learn to navigate complex contexts; they need to be attuned to self and others, and to co-configure hybrid environments in ways that enable joint distributed activity (Markauskaite & Goodyear, 2017). This requires a fundamental shift in how we conceptualise teachers’ digital competencies by moving from person-centred views to more holistic, ecological models. These models acknowledge the importance of teachers’ personal knowledge, skills, dispositions and other personal resources, but they also emphasise that the nature of professional work of university teachers is rather adaptive, distributed, relational, and entwined with collective knowledge practices, and so too are the capabilities needed for teaching. Therefore, digital technologies and competencies cannot be understood in isolation from a larger mix of tools, practices, goals, people, etc. that constitute teaching; and relationships between different elements and their digital and non-digital modalities are critical.

Further, the functionalist views of teachers’ roles and their digital competencies, which are often driven by the market-oriented goals of universities, need to be expanded. Sustainable universities need to address concerns of contemporary times. This requires teachers’ agentic engagement with a future-oriented, sustainable university mission, which is at its core postdigital.

## Data Availability

Data sharing not applicable to this article as no datasets were generated or analysed during the current study.

## Footnotes

1 Connell (2019) describes ‘sustainable’ as one of the five qualities of a good university. However, their notion of the good university echoes the notion of the sustainable university in a broader sense—the university that contributes to the sustainable development of the society at large.
